# Adipocytes induce distinct gene expression profiles in mammary tumor cells and enhance inflammatory signaling in invasive breast cancer cells

**DOI:** 10.1038/s41598-018-27210-w

**Published:** 2018-06-21

**Authors:** Annina Nickel, Christina Blücher, Omeir Kadri, Nancy Schwagarus, Silvana Müller, Michael Schaab, Joachim Thiery, Ralph Burkhardt, Sonja C. Stadler

**Affiliations:** 1 Institute of Laboratory Medicine, Clinical Chemistry and Molecular Diagnostics, University Hospital Leipzig, Leipzig, Germany; 2 LIFE Leipzig Research Center for Civilization Diseases, University of Leipzig, Leipzig, Germany

**Keywords:** Breast cancer, Cell biology

## Abstract

Obesity is a known risk factor for breast cancer. Since obesity rates are constantly rising worldwide, understanding the molecular details of the interaction between adipose tissue and breast tumors becomes an urgent task. To investigate potential molecular changes in breast cancer cells induced by co-existing adipocytes, we used a co-culture system of different breast cancer cell lines (MCF-7 and T47D: ER^+^/PR^+^/HER2^−^ and MDA-MB-231: ER^−^/PR^−^/HER2^−^) and murine 3T3-L1 adipocytes. Here, we report that co-culture with adipocytes revealed distinct changes in global gene expression pattern in the different breast cancer cell lines. Our microarray data revealed that in both ER^+^ cell lines, top upregulated genes showed significant enrichment for hormone receptor target genes. In triple-negative MDA-MB-231 cells, co-culture with adipocytes led to the induction of pro-inflammatory genes, mainly involving genes of the Nf-κB signaling pathway. Moreover, co-cultured MDA-MB-231 cells showed increased secretion of the pro-inflammatory interleukins IL-6 and IL-8. Using a specific NF-κB inhibitor, these effects were significantly decreased. Finally, migratory capacities were significantly increased in triple-negative breast cancer cells upon co-culture with adipocytes, indicating an enhanced aggressive cell phenotype. Together, our studies illustrate that factors secreted by adipocytes have a significant impact on the molecular biology of breast cancer cells.

## Introduction

The worldwide rising incidence of obesity poses a great burden to health care practitioners and the global health system. Obesity is not only a well-known risk factor for metabolic and cardiovascular diseases, but also accounts for approximately one-third of all new cancer diagnoses in the United States and for up to 20% of total cancer-related mortality^1,2^. There is increasing evidence linking obesity to elevated risk for several types of malignancies like breast, endometrial, colorectal and pancreatic cancer^1,2^. Several epidemiological studies demonstrate that obesity and excessive accumulation of adipose tissue are independent negative prognostic factors for breast cancer^3,4^. Although an increasing body of literature clearly demonstrates a link between increased body weight and tumor progression, the precise molecular mechanisms underlying this association remain elusive.

Adipose tissue mainly consists of mature adipocytes which are primarily responsible for energy homeostasis. However, there is accumulating evidence that their function is far more complex than just storing lipids. In fact, adipocytes also secrete cytokines, growth factors and adipokines and thereby influence other tissues in the body in a paracrine or endocrine manner^5^. Interestingly, numerous studies demonstrated that cytokines and adipokines such as IL-6, IL-1β, TNFα and Leptin are major factors in breast cancer progression^6^. Thus, adipose tissue may be an important modulator of breast cancer cell biology.

The systemic effects of obesity on cancer are mainly the consequence of adipocyte dysfunction^7^. In case of caloric excess over a longer period of time, adipocytes become hypertrophic and lose both metabolic function and the control over the release of pro-inflammatory cytokines, hormones, lipid metabolites and free fatty acids (FFA)^8^. A hallmark of dysfunctional adipose tissue is a chronic state of low-grade inflammation. The increased secretion of pro-inflammatory cytokines together with elevated lipid metabolites and FFAs support tumor progression by delivering essential building blocks and energy for cellular growth^9–11^. Importantly, several recent studies demonstrated that breast cancer cells and neighbouring adipocytes of the tumoral stroma also interact with each other directly^6,12^. This interaction leads to adipocytes with an activated, tumor supportive phenotype characterized by lipolysis, a decrease in adipocyte markers and an overexpression of pro-inflammatory cytokines like IL-6 and IL-1β. In turn, these so called cancer-associated adipocytes (CAA) contribute to the local inflammation and deliver energy for cell proliferation^13,14^. Together, these observations clearly point out that breast tumor cells are actively influencing the surrounding stroma to create an advantageous inflammatory microenvironment which, in turn, further supports tumor progression. However, detailed knowledge about which molecular pathways are activated in breast cancer cells upon interaction with adipocytes is still elusive. Here, we set up a co-culture system to study the effects of adipose tissue on breast cancer cells. Following co-culture with differentiated adipocytes, we profiled global gene expression changes in breast cancer cells. To our knowledge, this is the first study showing comprehensive microarray data of several breast cancer cell lines co-cultured with adipocytes. Our results demonstrate that adipocytes markedly affect gene expression profiles of co-cultured breast cancer cells. Specifically, we highlight the striking effects of adipocytes on triple negative breast cancer cells, which show a significant induction of pro-inflammatory genes and pathways upon co-culture in addition to an enhanced ability of cell migration and invasion.

## Results

### Co-culture with mature adipocytes affects distinct genes and signaling pathways in breast cancer cell lines, depending on the breast cancer subtype

To identify genes and underlying pathways in human breast cancer cells affected by interaction with mature adipocytes, two estrogen-receptor positive (ER^+^) breast cancer cell lines, MCF-7 and T47D, and the triple-negative (TN) breast cancer cell line MDA-MB-231 were co-cultivated with or without differentiated 3T3-L1 cells for the purpose of a microarray gene expression analysis. Murine 3T3-L1 preadipocytes can be induced chemically to differentiate into mature adipocytes and are a well-established adipocyte model for lipid metabolism and obesity research. The use of *in vitro* differentiated 3T3-L1 adipocytes allowed comparable experimental conditions for each of the co-culture experiments with human breast cancer cell lines (Supplemental Fig. 1). For co-cultivation analyses of 3T3-L1 and breast cancer cells, we set up a two-dimensional transwell system, which enables intercellular communication through soluble factors secreted into the medium but inhibits intermixture of the different cell types. Following 5 days of co-culture with or without differentiated adipocytes, total RNA was isolated from the human breast cancer cells and subjected to microarray gene expression analyses.

**Figure 1:**
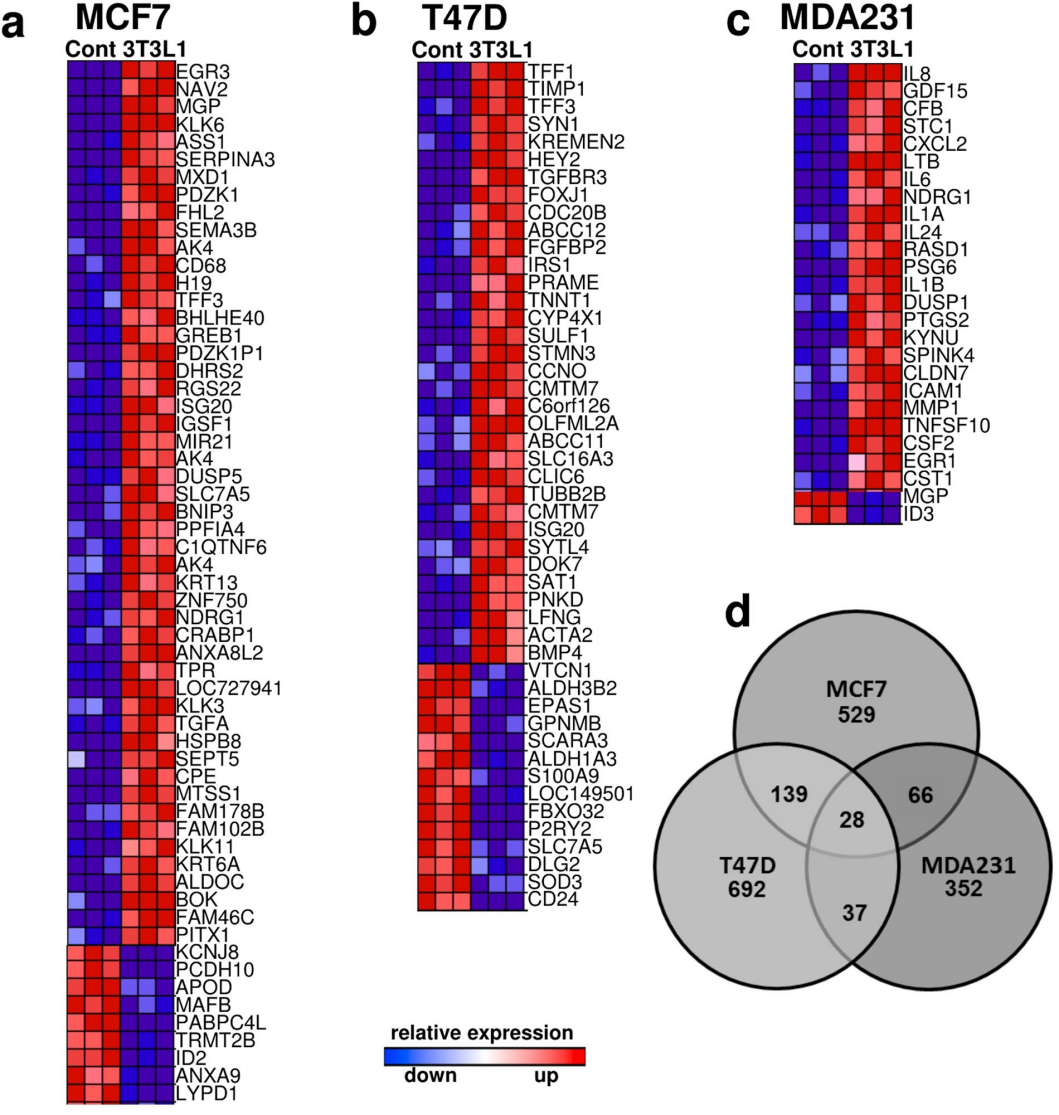
Distinct gene expression profiles in breast-cancer cell lines co-cultured with 3T3-L1 adipocytes. Rank-based heatmaps displaying the top up- and down-regulated genes (≥2.0-fold) in the breast cancer cell lines (**a**) MCF7, (**b**) T47D and (**c**) MDA-MB-231 co-cultured with 3T3-L1 adipocytes. Red indicates up-regulation in co-culture; blue indicates down-regulation; see Supplemental Tables S2–S4 for absolute fold-changes. (**d**) Venn-Diagram representation of significantly differentially expressed genes (≥1.3-fold expression differences) in the respective cell lines.

Data analysis revealed distinct sets of differentially expressed genes in ER+ and ER− breast cancer cells. We first filtered genes by applying a ≥2.0-fold change as cut-off value to identify the most up-and down-regulated genes (p < 0.05; FDR controlled) in response to co-cultivation with adipocytes (Fig. 1a–c; Supplemental Tables S2–S4).

In MCF-7 cells, 60 genes were differentially expressed by at least 2.0 fold following co-culture with adipocytes (51 up/9 down; Fig. 1a and Supplemental Table S2). Among up-regulated genes, 33% are genes known to be regulated by estrogen-signaling, such as *MGP*, *PLK1*, *PDZK1*, *TFF3 and GREB*^15–19^. Similarly, in the second ER + cell line T47D, 29% of ≥2.0-fold up- or down-regulated genes (48 total genes, 34 up/ 14 down; Fig. 1b and Supplemental Table S3) are regulated by estrogen-signaling, including *TFF1*, *TFF3*, *FOXJ1*, *IRS1 and BMP4*^18–27^. Gene expression analysis of the triple-negative (TN), invasive breast cancer cell line MDA-MB-231 revealed 26 genes (24 up/2 down) with at least 2.0-fold expression difference (Fig. 1c and Supplemental Table S4). Interestingly, the vast majority (67%) of these genes are involved in the inflammatory cell response, such as *IL8*, *CXCL2*, *IL6* or *IL1B*. Thus, interaction with adipocytes strongly induces several hormone-sensitive genes in the two ER^+^, non-invasive breast cancer cell lines MCF-7 and T47D, whereas in TN MDA-MB-231 cells, co-culture with adipocytes results in the induction of pro-inflammatory genes.

We next evaluated global gene expression signatures of MCF-7, T47D and MDA-MB-231 cells in response to co-culture with adipocytes by comparing all genes with at least ≥ 1.3-fold expression change. As shown in Fig. 1d, the majority of genes differed between the three respective breast cancer cell lines, but more genes overlapped between the two ER+ cell lines MCF-7 and T47D as compared to the TN MDA-MB-231 cells. We then annotated the unique gene lists from MCF-7, T47D and MDA-MB-231 cells and performed an enrichment analysis to identify significantly over- or under-represented Gene Ontology (GO) terms (Supplemental Figure S2 and Supplemental Table S5). Each cell line revealed a distinct pattern of enriched GO terms, but MCF-7 and T47D cells shared five out of nine, respectively twelve, significantly overrepresented GO terms in the biological process data set. In contrast, triple negative MDA-MB-231 cells do not overlap with the two ER+ cell lines in any of the significantly enriched GO terms. The most significantly enriched category in MDA-MD-231 cells was “response to stress” (Figure S2 and Supplemental Table S5).

To enhance the information about which signaling pathways might be affected in MDA-MB-231 cells by co-culture with adipocytes, we performed a gene-set over-representation analysis using ConsensusPathDB. This gene set analysis included all genes found to be differentially expressed by at least 1.3 fold (n = 199) as compared to the control. Our analysis revealed that up-regulated genes are significantly over-represented in inflammation-related pathways, such as TNF signaling, NF-kB signaling and cytokines and inflammatory response (Table 1 and see Supplemental Table S6 for more details). Down-regulated genes in MDA-MB-231 cells showed a significant association with cell cycle related pathways such as Mitotic Cell Cycle and Mitotic G1-G1/S phases (Table 2 and see Supplemental Table S7 for more details). Thus, the pathway analysis substantiates that a vast majority of the strongest up-regulated genes are genes involved in inflammatory processes, while down-regulated genes were associated with cell cycle regulation.

**Table 1:** Over-represented cellular pathways of up-regulated genes in MDA-MB-231 cells grown as a co-culture with 3T3-L1 adipocytes.

Pathway name	set size	overlapping genes (% of total genes in pathway)	p-value	database
TNF signaling pathway	110	17 (15.5%)	7.5e-12	KEGG
Rheumatoid arthritis	90	15 (16.9%)	3.69e-11	KEGG
NF-kappa B signaling pathway	95	14 (14.9%)	9.28e-10	KEGG
Spinal Cord Injury	117	14 (12.0%)	1.71e-08	Wikipathways
Senescence and Autophagy in Cancer	105	13 (12.4%)	3.72e-08	Wikipathways
Nuclear Receptors Meta-Pathway	316	22 (7.0%)	4.01e-08	Wikipathways
Glucocorticoid Receptor Pathway	71	11 (15.5%)	4.02e-08	Wikipathways
HTLV-I infection	258	19 (7.4%)	1.47e-07	KEGG
Photodynamic therapy-induced HIF-1 survival signaling	36	8 (22.2%)	1.62e-07	Wikipathways
AGE-RAGE signaling pathway in diabetic complications	101	12 (11.9%)	2.00e-07	KEGG
Selenium Micronutrient Network	83	11 (13.3%)	2.11e-07	Wikipathways
HIF-1-alpha transcription factor network	67	10 (14.9%)	2.43e-07	PID
Cytokines and Inflammatory Response	29	7 (25.0%)	4.15e-07	Wikipathways
AP-1 transcription factor network	71	10 (14.1%)	4.26e-07	PID

Over-represented cellular pathways of ≥1.3-fold up-regulated genes in MDA-MB-231 breast cancer cells grown as co-cultures with 3T3-L1 adipocytes for 5 days. The Consensus Path Database over-representation analysis was used including all available databases (accessed 25. April 2017).

**Table 2:** Over-represented cellular pathways of down-regulated genes in MDA-MB-231 cells grown as a co-culture with 3T3-L1 adipocytes.

Pathway name	set size	overlapping genes (% of total genes in pathway)	p-value	database
Cell Cycle, Mitotic	468	35 (7.5%)	2.95e-20	Reactome
Cell Cycle	551	37 (6.7%)	7.96e-20	Reactome
Retinoblastoma (RB) in Cancer	89	14 (15.7%)	5.92e-13	Wikipathways
M Phase	267	19 (7.1%)	6.79e-11	Reactome
DNA Replication	60	10 (16.7%)	7.56e-10	Reactome
S Phase	82	11 (13.4%)	1.16e-09	Reactome
Activation of the pre-replicative complex	32	8 (25.0%)	1.3e-09	Reactome
Mitotic Anaphase	135	13 (9.6%)	2.24e-09	Reactome
Mitotic Metaphase and Anaphase	136	13 (9.6%)	2.46e-09	Reactome
Mitotic G1-G1/S phases	92	11 (12.0%)	4.07e-09	Reactome
DNA Replication Pre-Initiation	37	8 (21.6%)	4.55e-09	Reactome
M/G1 Transition	37	8 (21.6%)	4.55e-09	Reactome
Synthesis of DNA	55	9 (16.4%)	6.54e-09	Reactome
DNA Replication	42	8 (19.0%)	1.33e-08	Wikipathways
Cell Cycle	103	11 (10.7%)	1.36e-08	Wikipathways

Over-represented cellular pathways of ≥1.3-fold down-regulated genes in MDA-MB-231 breast cancer cells grown as co-cultures with 3T3-L1 adipocytes for 5 days. The Consensus Path Database over-representation analysis used including all available databases (accessed 25. April 2017).

### Confirmation of microarray data by quantitative Real-Time PCR (qRT-PCR)

To validate the data of our microarray analysis, several of the most up- and down-regulated candidate genes in each breast cancer cell line were tested by qRT-PCR. Further, to determine if a sustained interaction of adipocytes and tumor cells was a requirement for the observed changes in the gene expression profiles of the breast cancer cell lines, breast cancer cells were either co-cultured with adipocyte-conditioned medium (ACM), or with 3T31-L1 adipocytes (CoAd). Moreover, to evaluate the impact of undifferentiated pre-adipocytes, cancer cells were co-cultured with undifferentiated 3T3-L1 fibroblasts (CoFi) as well. Cancer cells grown in regular growth medium served as controls. In T47D cells, qRT-PCR confirmed a significant up-regulation of the genes *TFF1*, *TIMP1* and *TGFBR3* (Supplemental Fig. 3a) upon co-culture with 3T3-L1 adipocytes (CoAd). Likewise, the genes *VTCN1*, *EPAS1*, *GPNMB* and *CD24* were verified to be down-regulated (Supplemental Fig. 3b). Similar results were retrieved for MCF-7 cells, with a significant up-regulation of the genes *NAV2*, *EGR3* and *SERPINA3* and down-regulation of *MAFB*, *PCDH10* and *PABPC4L* under all mentioned culture conditions (Supplemental Fig. 4a,b). While in most cases co-culture with adipocytes led to the strongest up-or down-regulated of the selected genes, also co-culture with ACM often induced significant changes in gene expressions. These results are in line with accumulating clinical and experimental evidence showing that complex interactions between tumor cells and cells of the surrounding stroma exist and that factors secreted by adipocytes are involved in changing gene expression patterns of ER^+^ breast cancer cells.

**Figure 2:**
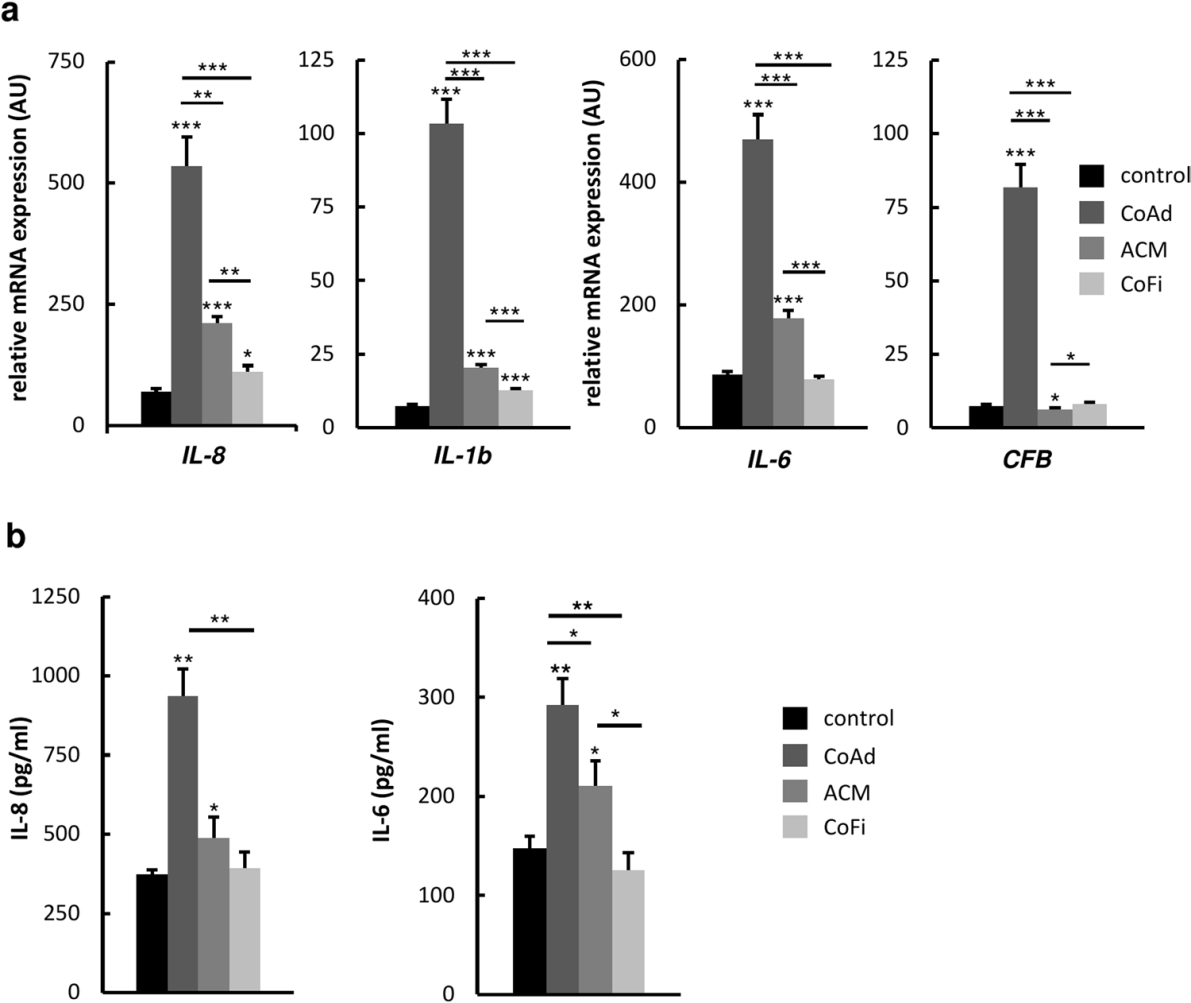
MDA-MB-231 cells show increased expression of pro-inflammatory genes and enhanced IL-8 and IL-6 secretion upon co-culture with differentiated 3T3-L1 adipocytes. MDA-MB-231 breast cancer cells were co-cultured either with 3T3-L1 adipocytes (CoAd), 3T3-L1 fibroblasts (CoFi), Adipocyte-conditioned medium (ACM) or alone (control) for 5 days. (**a**) After 5 days of co-culture, total RNA was extracted. Relative mRNA expression of selected genes was determined by performing quantitative RT-PCR. (**b**) Following 5 days of co-culture, supernatants were collected and cytokine expression was determined using ELISA Kits specifically detecting human IL-8 and IL-6. Data are presented as the means ± SD of triplicates from one representative experiment. Significance is relative to control unless indicated otherwise (*p < 0.05; **p < 0.005; ***p < 0.0005).

Corroborating the microarray analysis, we also detected significant up-regulation of pro-inflammatory genes *IL8*, *IL6*, *IL1B* and *CFB* in MDA-MB-231 cells co-cultured with adipocytes (Fig. 2a). Interestingly, co-culture with 3T3-L1 adipocytes consistently led to the strongest up-regulations of the examined genes in MDA-MB-231 cells, whereas co-culture with ACM or 3T3-L1 fibroblasts resulted in smaller or no effects, respectively (Fig. 2a). These data indicate, that the strongest changes of expression levels of the mentioned genes are induced by the constant presence of differentiated adipocytes during the course of co-culture, most likely through the continuous, active exchange of secreted factors.

### Interaction with adipocytes increases secretion of IL-8 and IL-6 by MDA-MB-231 cells

Interestingly, two of the most up-regulated genes in the TN breast cancer cell line MDA-MB-231 following co-culture with adipocytes were *IL8* and *IL6*, encoding the pro-inflammatory cytokines IL-8 and IL-6. This was of special interest, since interleukins, such as IL-8 and IL-6, are considered to be metastatic factors in breast cancer progression^9–14,28^. To test, whether the elevated mRNA expression levels of *IL8* and *IL6* would also translate into increased secretion of these cytokines by MDA-MB-231 cells, we determined human IL-8 and IL-6 levels in cell culture supernatants with ELISA kits specifically detecting human IL-8 and IL-6. Consistently, secreted levels of IL-8 and IL-6 originating from the human MDA-MB-231 cells were significantly increased in co-cultured cells as compared to controls (Fig. 2b). Cultivation of MDA-MB-231 cells with ACM also led to a significant increase of IL-8 and IL-6 secretion, but to a much lesser extent. In comparison, interaction with undifferentiated 3T3-L1 fibroblasts did not affect the cytokine secretion levels of MDA-MB-231 cells. These results indicate that the production of IL-8 and IL-6 in MDA-MB-231 cells is specifically enhanced by factors secreted from adipocytes and is even more pronounced if adipocytes are present over the entire course of co-cultivation. In addition, we determined murine IL-6 levels in cell culture supernatants (Supplemental Fig. 5). Interestingly, co-culture of adipocytes and breast cancer cells resulted in a drastic increase of murine IL-6 levels. Co-culture of breast cancer cells with fibroblasts also led to an increase of murine IL-6 from fibroblasts, but to a much lesser extent. In the supernatant of MDA-MB-231 cells alone no murine IL-6 was detectable. Together these results indicate that breast cancer cells provoke increased IL-6 secretion from adipocytes which probably feeds into the pro-inflammatory signaling loop of the breast cancer cells. To date, there are several lines of evidence indicating that tumor cells induce surrounding cells of the tumor stroma such as macrophages^29^, fibroblasts and adipocytes^13,14^ to secrete cytokines and other factors to create a tumor-supporting micromilieu^9^. Our data here demonstrate for the first time, that the crosstalk between breast cancer cells and adipocytes also changes the expression profile of the tumor cells towards a pro-inflammatory phenotype.

### Co-culture with adipocytes enhances NF-κB signaling in MDA-MB-231 cells

**Figure 3:**
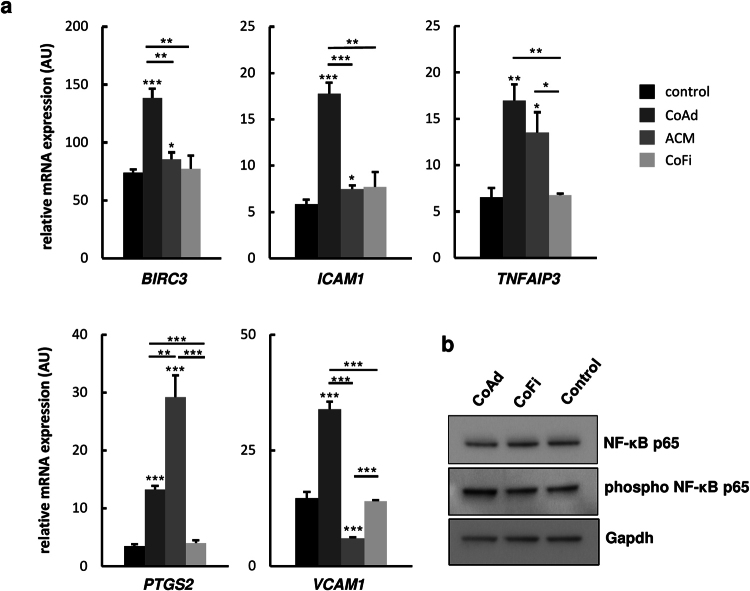
Increased p65 phosphorylation and mRNA expression of NF-κB target genes in co-cultured MDA-MB-231 cells. MDA-MB-231 breast cancer cells were co-cultured either with 3T3-L1 adipocytes (CoAd), 3T3-L1 fibroblasts (CoFi), Adipocyte-conditioned medium (ACM) or alone for 5 days. (**a**) mRNA expression levels of selected NF-κB-response genes were analysed by qRT-PCR analyses. Data are presented as the means ± SD of triplicates from one representative experiment. Significance is relative to control unless indicated otherwise (*p < 0.05; **p < 0.005; ***p < 0.0005). (**b**) Representative immunoblots of indicated whole cell extracts of MDA-MB-231 cells probed with antibodies against NF- κB p65, phosphorylated-NF- κB p65 and Gapdh (loading control). Blots’ images where cropped to show relevant areas.

**Figure 4:**
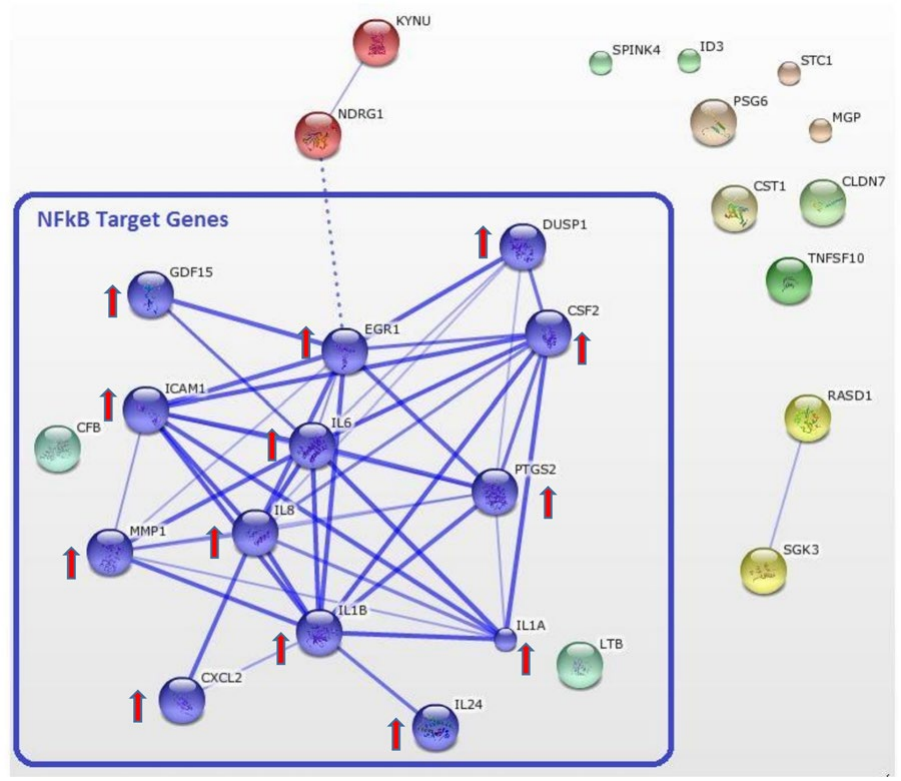
STRING pathway analysis of up- and downregulated genes in MDA-MB-231 cells co-cultured with adipocytes. STRING pathway analysis of genes that were up- or down-regulated ≥2.0 fold in MDA-MB-231 cells upon co-culture with 3T3-L1 adipocytes (genes listed in Supplemental Table S4). The identified interactions of the gene products revealed a cluster of NF-κB target genes. Thicker lines are indicative for higher confidence of interaction.

In view of the up-regulated pro-inflammatory genes, e.g. *IL6* and *IL8*, and signaling pathways that can be assigned to NF-κB signaling (see Table 1), we next wanted to examine if this pathway was in fact affected in MDA-MB-231 cells upon co-culture with differentiated adipocytes. Quantitative RT-PCR analysis of well-established NF-κB target genes *BIRC3*, *ICAM1*, *TNFAIP3*, *PTGS2* and *VCAM1*, showed significantly higher expression levels of all of these genes in MDA-MB-231 cells co-cultivated with 3T3-L1 adipocytes as compared to breast cancer cells grown in presence of 3T3-L1 fibroblasts, ACM or regular growth medium (Fig. 3a). Moreover, Western blot analysis of MDA-MB-231 whole cell extracts showed increased levels of phospho-NF-κBp65 in breast cancer cells co-cultured with adipocytes as compared to breast cancer cells co-cultured with fibroblasts or control medium whereas the levels of NF-κBp65 were comparable in all three samples (Fig. 3b). These data demonstrate that co-culture with adipocytes increases active NF-κB signaling in MDA-MB-231 breast cancer cells. In addition, we applied STRING for an interaction analysis performed with all genes showing ≥2.0-fold expression changes (n = 26) in MDA-MB-231 cells cultured with adipocytes. This analysis revealed a prominent cluster of interacting NF-κB signaling molecules (Fig. 4). 15 (57.7%) out of 26 included genes allocated to the NF-κB pathway, indicating that this network plays an important role in the response of MDA-MB-231 cells to stimuli produced by co-cultured adipocytes.

### Activation of IL-8 in MDA-MB-231 cells co-cultured with adipocytes is dependent on active NF-κB-signaling

**Figure 5:**
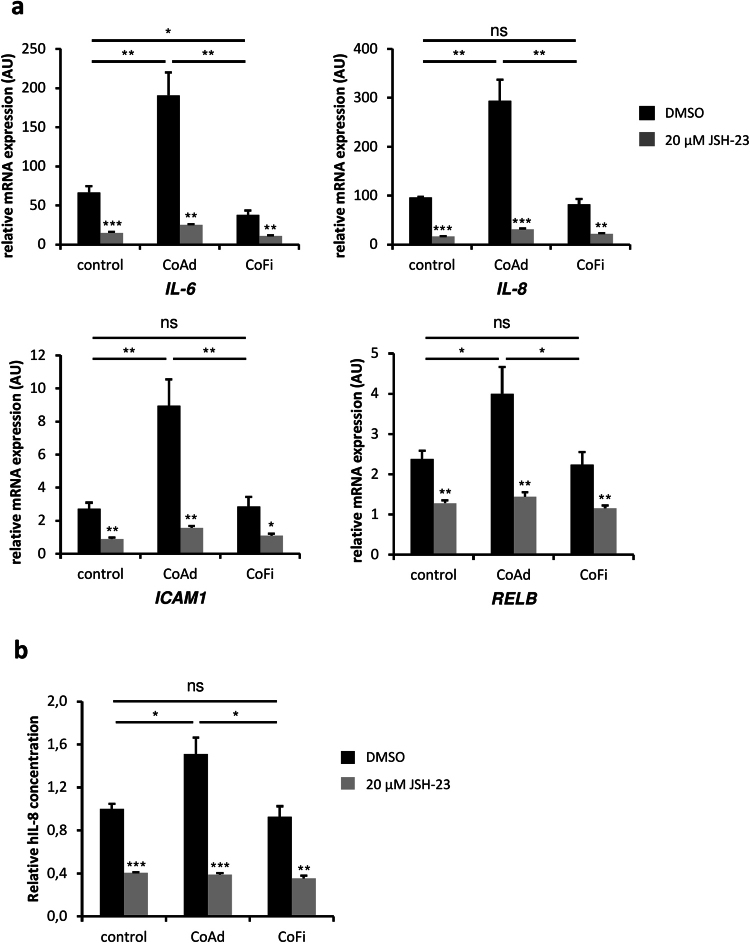
Increased expression of NF-kB target genes and secretion of hIL-8 is dependent on active NF-KB signaling in MDA-MB-231 breast cancer cells. **(a)** MDA-MB-231 breast cancer cells were co-cultured either with 3T3-L1 adipocytes (CoAd), 3T3-L1 fibroblasts (CoFi) or alone (control) for 5 days in presence or absence of 20 µM JSH-23. mRNA expression levels of selected NF-κB-target genes were analysed by qRT-PCR analyses. (**b**) Following 5 days of co-culture with or without 20 µM of JSH-23 present, supernatants were collected and cytokine levels were determined using ELISA Kit specifically detecting human IL-8. Data are presented as the means ± SD of triplicates from one representative experiment. Significance is relative to basal conditions (JSH-23 vs DMSO) unless indicated otherwise (*p < 0.05; **p < 0.005; ***p < 0.0005).

Given that co-culture with adipocytes led to increased expression levels of putative NF-κB target genes and increased IL-8 secretion, we performed co-culture experiments of MDA-MB-231 cells and adipocytes in the presence or absence of the NF-κB-inhibitor JSH-23. To examine if inhibition of NF-κB signaling directly affects the expression levels of downstream targets, quantitative RT-PCR analysis of established NF-κB target genes *ICAM1*, *IL6*, *IL8* and *RELB* was performed. The results showed significantly higher expression levels of these genes in MDA-MB-231 cells co-cultivated with 3T3-L1 adipocytes as compared to breast cancer cells grown in presence of 3T3-L1 fibroblasts or regular growth medium (Fig. 5a). Interestingly, addition of JSH-23 to co-cultures significantly reduced the expression levels of the mentioned genes. Next, to determine if inhibition of NF-κB-signaling would also affect the secretion of IL-8 from MDA-MB-231 cells, we analyzed secreted IL-8 levels by ELISA. Consistent with the results described above (Fig. 2a,b), secreted levels of IL-8 originating from the human MDA-MB-231 cells were significantly increased in breast cancer cells co-cultured with adipocytes as compared to controls or co-culture with fibroblasts (Fig. 5b). Interaction with undifferentiated 3T3-L1 fibroblasts did not affect the cytokine secretion levels of IL-8 from MDA-MB-231 cells as compared to the medium only control. However, addition of JSH-23 (20 µM) significantly reduced the levels of secreted IL-8 in all treatment conditions, indicating that secretion of IL-8 is dependent on active NF-κB-signaling in MDA-MB-231 breast cancer cells.

### Interaction with mature adipocytes increases the motility of MDA-MB-231 cells

**Figure 6:**
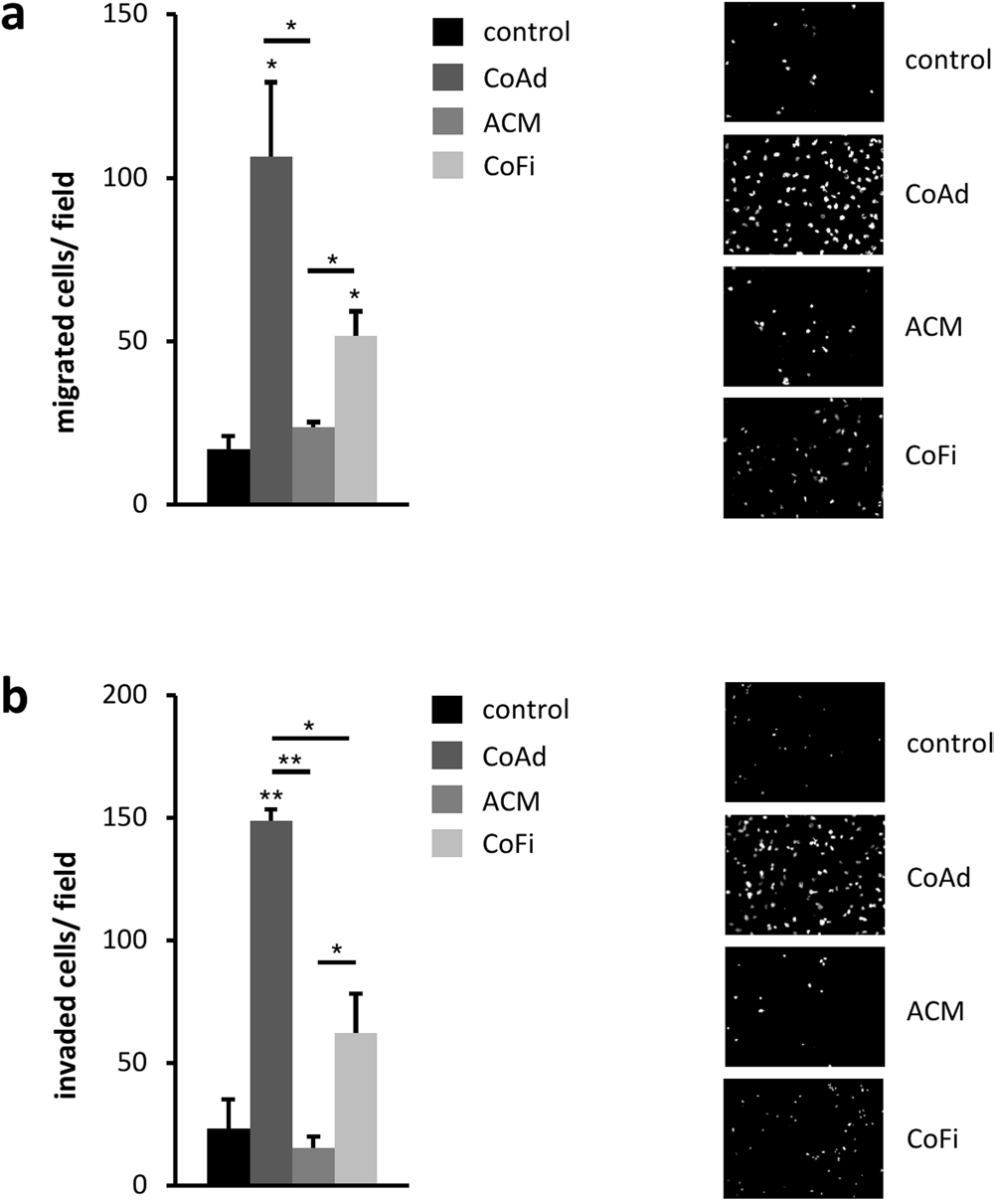
Co-Culture with 3T3-L1 adipocyte significantly increases the migratory abilities of MDA-MB-231 breast cancer cells. (**a**) Breast cancer cells were seeded into the upper chamber of a transwell-system (8 µM pore size) with either 3T3-L1 adipocytes (CoAd), 3T3-L1 fibroblasts (CoFi) or adipocyte-conditioned medium (ACM) in the bottom well. DMEM complemented with 10% FCS served as control. (**b**) For invasion assays, breast cancer cells were seeded in tissue culture inserts coated with Matrigel. Following 21 h of co-culture, cells that had transversed to the lower surface of the membrane were fixed with 95% ethanol and cell nuclei were stained with DAPI and counted. Values represent the mean of duplicates ± SD of one representative experiment. Significance is relative to control unless indicated otherwise (*p < 0.05; **p < 0.005).

In cancer cells, activation of inflammatory pathways such as NF-κB-signaling, either induced by acute inflammatory processes or constitutively activated, can induce a pro-tumorigenic response, such as cell survival, controlling epithelial-mesenchymal-transition and metastasis^30^. Given the robust induction of pro-inflammatory signaling pathways in response to interaction with adipocytes, we sought to evaluate the motility of MDA-MB-231 cells using transwell migration and invasion assays. Interestingly, MDA-MB-231 cells co-cultured with mature adipocytes showed significantly more migration and invasion than MDA-MB-231 cells (co-) cultured with undifferentiated preadipocytes, ACM or medium alone (Fig. 6). Thus, our findings demonstrate that interaction with adipocytes induces functional changes in MDA-MB-231 cells consistent with increased cell migration and invasion, important steps in cancer progression and metastasis. Taken together, our data show that interaction with adipocytes induces pro-inflammatory responses in breast cancer cells, in this case MDA-MB-231 cells, a fact that has been underappreciated thus far. In addition, we demonstrate that co-cultivation with adipocytes increases the motility of these TN breast cancer cells. These results are in line with the notion that activation of inflammatory pathways can enhance the aggressiveness of cancer cells^31^.

## Discussion

Despite a growing body of evidence underlining the important role of obesity and excess adipose tissue in survival and growth of breast tumors, detailed knowledge about the molecular mechanisms linking adipocytes to tumor growth, survival, and metastasis is limited. We hypothesized that interaction with adipocytes leads to the induction of pro-tumorigenic genes in breast cancer cells, since adipocytes have been described to exert several tumor promoting effects on cancer cells^13,14,32,33^.

To investigate potential molecular changes in breast cancer cells, induced by co-existing adipocytes, we established a two-dimensional co-culture system of three different human breast cancer cell lines (T47D; MCF-7: ER^+^/PR^+^/HER2^−^; MDA-MB-231: ER^−^/PR^−^/HER2^−^) and murine 3T3-L1 adipocytes. Following co-culture, global gene expression in cancer cells was profiled by microarrays. To our knowledge, this is the first study showing comprehensive, genome-wide gene expression analyses in hormone receptor positive and negative breast cancer cells following co-culture with adipocytes. Our microarray data revealed changes in gene expression pattern in response to adipocytes, unique for each breast cancer cell line. However, the two ER positive cell lines T47D and MCF-7 shared the induction of several hormone-sensitive genes upon co-culture with adipocytes, whereas the invasive breast cancer cell line MDA-MB-231 showed a significant inflammatory response. Several studies have shown, that obesity is a risk factor for the development of hormone receptor positive breast cancer, especially in postmenopausal women^34–36^. Aromatization of androgens (androstenedione and testosterone) to estrogens (estradiol and estrone) is catalyzed by the aromatase. Whereas in premenopausal women the highest levels of aromatase are found in granulosa cells of the ovaries, adipose tissue becomes the major source of aromatase after menopause^37^. Estrogen promotes tumor growth by binding to its receptors on ER+ tumor cells and stimulating the expression of estrogen responsive genes. In our adipocyte co-culture experiments approximately one third of the differentially expressed genes (≥2.0-fold) in the two hormone receptor positive breast cancer cell lines MCF-7 and T47D are hormone sensitive. These results are in line with the current understanding that ER+ breast cancer cells are strongly influenced by estrogen derived from adipose tissue, which is considered to be a major contributor to tumor proliferation and progression by activating pro-tumorigenic estrogen responsive genes such as TFF1/pS2, Cyclin D1 or c-myc^21,38–41^.

The present knowledge concerning the role of adipose tissue in progression of triple negative breast cancer is far more limited. Triple negative breast cancer is associated with an aggressive pathology, a cancer diagnosis at younger ages and poorer survival^42–44^. Study reports regarding the relationship between BMI of breast cancer patients and the risk of triple negative breast cancer are rare and inconsistent. Whereas some studies reported a positive association between BMI and risk of postmenopausal triple negative breast cancer^45–47^ other studies found no correlation between BMI and postmenopausal triple-negative breast cancer^45,48^. However, a large number of studies demonstrated that adipose tissue in obesity is in a state of chronic low-grade inflammation as evidenced by the presence of immune cells such as lymphocytes and macrophages. Further, cytokines secreted by adipose tissue like IL-1β, IL-6 and IL-8 have been implicated in tumor progression by stimulating tumorigenic pathways in cancer cells, such as TNFα and NF-κB signaling. In addition to systemic effects of adipose tissue dysfunction, several recent studies demonstrated that dysfunctional adipose tissue exerts similar effects on tumor cells growing in their direct vicinity and vice versa^6,12^. For instance, it has been shown that cancer cells induce changes in neighbouring adipocytes thereby creating activated fat cells called cancer-associated adipocytes (CAA). These CAAs are characterized by increased lipolysis, a decrease in adipocyte markers and an overexpression of pro-inflammatory cytokines like IL-6 and IL-1β. Conversely, these changes in the adipocytes contribute to the local inflammation and in turn drive tumor progression, among other mechanisms by promoting migration and invasion capabilities of breast cancer cells^13^. Here, in our study we present data showing that the interaction of adipocytes and breast cancer cells, not only stimulates the secretion of inflammatory cytokines in adipocytes, but also in the breast cancer cells themselves. A fact that has been underappreciated so far, since most of the conducted adipocyte-breast cancer cell co-culture studies focused on the up-regulation and secretion of cytokines induced in interacting adipocytes. In case of the TN MDA-MB-231 cells studied here, co-culture with adipocytes led to the induction of inflammatory pathways, such as NF-κB and TNFα, and elevated secretion levels of IL-8 and IL-6. Interestingly, expression levels of *IL8* and *IL6* have been shown to be inversely correlated to the estrogen receptor status, with high *IL8* and *IL6* expression in ER^−^ breast cancer cell lines with high invasive potential^49,50^.

There is increasing evidence that NF-κB signaling is an important player in cancer development and progression^23^. NF-κB signaling in cancer cells can be activated by factors such as IL-1β, TNFα and IL-8, IL-6 and MCP-1 secreted by cells in the microenvironment of the tumor, e.g. macrophages or adipocytes^51–53^. NF-κB, in turn, is known to induce TNFα, IL-1, IL-6 and IL-8 and thereby contributing to tumor cell growth an proliferation^52^. Moreover, NF-κB signaling contributes to cancer progression by regulating epithelial to mesenchymal transition and metastasis^54^. These observations are in agreement with the results of our study. Next to the increased expression and secretion of IL-6 and IL-8 from MDA-MB-231 cells upon co-culture with adipocytes, pathway and STRING analyses of differentially expressed gene sets also indicated an activation of the NF-κB signaling pathway. This was further corroborated by expression analyses of NF-κB target genes revealing significant upregulation of these genes in MDA-MB-231 cells following co-culture with adipocytes. Interestingly, co-culture experiments with the specific NF-KB inhibitor JSH-23 drastically decreased these effects. We also found that co-culture with differentiated 3T3-L1-adipocytes significantly increased the migratory abilities of MDA-MB-231 cells by performing transwell migration and invasion assays. This observation is in line with findings from other groups demonstrating that interaction with adipocytes or adipocyte-conditioned medium enhances the ability of migration and invasion of cancer cells^10,12,55^. It is very conceivable that one of the driving forces in increasing cell migration and invasion here are pro-inflammatory cytokines, like IL-6 and IL-8, secreted from mature adipocytes, which are known to play a role in breast cancer progression. In our work here, we demonstrate that co-culture with differentiated adipocytes not only induces IL-6 and IL-8 gene expression and production within TN breast cancer cells, but also increases murine IL-6 production of the adipocytes. This fits the hypothesis that factors secreted from co-cultivated adipocytes could feed into the autocrine production loop of IL-8 and IL-6 in MDA-MB-231 cells, thereby potentially promoting a more aggressive phenotype of these cells for example by enhancing NF-κB signaling. To suspend this positive feedback loop of inflammation between breast tumor cells and their surrounding microenvironment seems to be a highly considerable treatment strategy, especially for the treatment of triple-negative breast carcinomas, which currently only have limited targeted therapeutic options. Therapeutics specifically inhibiting IL-6, IL-8 and NF-κB signaling could be a promising treatment option for obese women with triple-negative breast cancer.

## Materials and Methods

### Cell culture of breast cancer cell lines

Human breast carcinoma cell lines MDA-MB-231, MCF-7 and T47D were obtained from the American Type Culture Collection (ATCC). The murine pre-adipocyte cell line 3T3-L1 was kindly provided by Jan L. Breslow (The Rockefeller University, New York). MDA-MB-231, MCF-7 and 3T3-L1 cells were cultured in Dulbecco’s modified Eagle’s medium (DMEM) and Roswell Park Memorial Institute (RPMI) 1640 Medium (T47D) supplemented with 10% FCS (Biochrom GmbH-Millipore) and 1% Antibiotic-Antimycotic (Gibco®). T47D cells were grown in Roswell Park Memorial Institute (RPMI) 1640 Medium supplemented with 10% FCS (Biochrom GmbH-Millipore) and 1% Antibiotic-Antimycotic (Gibco®). All cell cultures were maintained at 37 °C in 5% CO_2_.

### *In vitro* differentiation of 3T3-L1 preadipocytes

Differentiation of 3T3-L1 preadipocytes into mature adipocytes was performed as previously described^56^. In brief, 2-day postconfluent cells (designated day 0) were treated with DMEM, 10% FCS, 1% Antibiotic-Antimycotic (Gibco®), 0.5 mM 3-isobutyl-1-methylxanthine (IBMX), 0.25 µm Dexamethasone, 1 µg/ml insulin and 2 µM Rosiglitazone (all Sigma-Aldrich) to induce differentiation. After 3 days, medium was replaced by DMEM, 10% FCS, 1% Antibiotic-Antimycotic (Gibco®) and 1 µg/ml insulin. On day 6, medium was replaced with regular culture medium (DMEM, 10% FCS, 1% Antibiotic-Antimycotic), and further exchanged every 2-3 days. 3T3-L1 adipocytes were used for co-cultures with human breast cancer cells from day 10 to 18 after starting differentiation. 3T3-L1 preadipocyte control cells (undifferentiated control) were maintained in DMEM supplemented with 10% FCS and 1% Antibiotic-Antimycotic (Gibco®).

To generate adipocyte-conditioned medium (ACM), 3T3-L1 adipocytes (between day 10 and 18 after starting differentiation) were cultured in DMEM, 10% FCS, 1% Antibiotic-Antimycotic (Gibco®) for 48 h. ACM was then collected and filtered using a 0.8 µm syringe filter to remove any cellular debris.

### Co-culture model

Murine 3T3-L1 adipocytes and human breast cancer cells were co-cultured using ThinCert™ cell culture inserts (0.4 µm pore size; Greiner BioOne) in 6-well tissue culture plates (Greiner BioOne), filled with regular growth medium. Breast cancer cells were seeded in the top chamber of the co-culture system with adipocytes (CoAd) or without (control) adipocytes in the bottom well. To analyze the effect of undifferentiated 3T3-L1 fibroblasts (preadipocytes) and adipocyte-conditioned medium on tumor cells, breast cancer cells were cultured with either fibroblasts (CoFi) or adipocyte-conditioned medium (ACM) in the bottom chamber. Inhibition of NF-κB was performed with 20 µM JSH-23 (Selleckchem) dissolved in DMSO.

### RNA isolation, cDNA synthesis and quantitative RT-PCR

For RNA isolation breast cancer cells were co-cultured with adipocytes (CoAd), fibroblasts (CoFi), ACM or by themselves (control) for 5 days. Total RNA was isolated using the QIAGEN RNeasy Micro Kit according to the manufacturer’s instructions (QIAGEN). First strand cDNA was synthesized from 2 µg of each total RNA sample using Superscript II reverse transcriptase (Invitrogen). Quantitative RT-PCRs were performed using EvaGreen™ Mastermix (Solis BioDyne, Estonia) on ViiA 7 thermocycler equipment (Applied Biosystems). The relative expression of each gene was determined using the comparative CT method (ΔΔCt) and glyceraldehyde 3-phosphate dehydrogenase (GAPDH) as housekeeping gene^57^. Each experiment was conducted with triplicates and replicated at least three times. All primer sequences used for RT-PCR analysis are listed in Supplemental Table 1.

### Microarray analysis

RNA integrity was assessed with the Agilent 2100 Bioanalyzer (Agilent Technologies Inc., Palo Alto, California). cRNA labeling and hybridization to the Illumina HT-12 v4 expression bead chips was carried out at the DNA Technologies core unit of the Center of Clinical Research (IZKF), University Hospital Leipzig. All steps were performed according to the manufacturer’s specifications. Microarrays were scanned on an iScan array scanner (Illumina) and raw array data were processed and background subtracted in Illumina GenomeStudio. Further analysis was performed using the Chipster open source platform^58^. Expression values were quantile normalized and log2-transformed using the Bioconductor package ‘lumi’ implemented in Chipster^59^. Statistical comparison between the sample groups was done within Chipster using the empirical Bayes method^60^ and the Benjamini-Hochberg (BH) multiple-testing correction of the raw p-values. FDR threshold of 5% (q < 0.05) was used for filtering differentially expressed genes in each condition. The microarray data have been deposited in the ArrayExpress database at EMBL-EBI (www.ebi.ac.uk/arrayexpress) under accession number E-MTAB-6855.

### Panther Gene Ontology

We used the gene ontology (GO) enrichment tool from the PANTHER Classification System accessible at http://pantherdb.org (Version 13.1) to evaluate characteristics of sets of genes. Genelists from the respective breast cancer cell lines (MCF7, T47D, MDA231) included all genes with at least 1.3-fold expression changes upon co-culture with 3T3-L1 adipocytes and were tested for overrepresentation (PANTHER Overrepresentation Test (Released 20171205) in the PANTHER GO-slim Biological Process data set. The complete homo sapiens gene list was used as reference set to test for overrepresentation. Fisher’s exact test with FDR multiple test correction was applied and p < 0.05 was considered significant.

### Pathway analysis with ConsensusPathDB

Enrichment analysis was performed employing ConsensusPathDB (release 30), of the Max Planck Institute for Molecular Genetics in Berlin, Germany, by using the overrepresentation analysis online tool (http://consensuspathdb.org/). To perform gene set analysis, genes that were found to be up- or down-regulated by at least 1.3-fold in breast cancer cells upon co-culture with 3T3-L1 adipocytes, were included. Up-and down-regulated genes were analyzed separately. We searched for pathways as defined by PID, NetPath, Biocarta, Reactome, Wikipathways and KEGG, with a minimal overlap with the input list of 2 and a p-value cutoff at 0.01.

### String Database Analysis

The STRING database (Search Tool for the Retrieval of Interacting Genes/Proteins (http://string-db.org)) (Version 10) was used to visualize interactions among differentially expressed genes^61^.

### Migration and Invasion Assays

Migration Assays were performed using ThinCert™ Cell culture inserts (8 µM pore size; 6-well plate) by Greiner Bio-One. For Invasion assays BD BioCoat™ Matrigel™ Invasion Chambers (8 µM pore size; BD Biosciences) were used. For both assays, MDA-MB-231 cells were resuspended in serum-free DMEM and seeded into the cell culture inserts at a cell density of 3 × 10^5^ cells/well (Migration Assays) and 5 × 10^4^ cells/well (Invasion Assays), respectively. The bottom chambers contained either 3T3-L1 adipocytes (CoAd), 3T3-L1 fibroblasts (CoFi), ACM or regular growth medium (DMEM, 10% FCS, 1% AA) as medium control. After 21 h of incubation, cell culture inserts were removed and membranes were fixed with 95% ethanol for 30 sec. The upper side of the membrane was wiped with cotton swabs to remove non-migrated cells. Membranes were then rinsed with clean water, cut out of the insert and mounted on slides with ProLong® Gold Antifade reagent containing DAPI (Life Technologies) to stain nuclei. Microscopic images were taken (five optical fields/membrane, 20 or 40x magnification) using a Zeiss Apotome Fluorescence Microscope. The values for cell migration and invasion were calculated as the mean of migrated and invaded cells/field. Experiments were performed in duplicates and repeated at least three times with consistent results.

### ELISA

The concentrations of secreted human IL-6 (hIL-6) and IL-8 (hIL8) in cell culture supernatants were determined using human IL-6 and IL-8 ELISA Kits (MesoScale Discovery) as recommended by the manufacturer. The ELISA assays were evaluated for cross-reactivity with mouse IL-6 or KC/GRO (the mouse homologue for human IL-8) by the manufacturer. No significant cross-reactivity was observed. Mouse IL-6 (m IL-6) was detected using a ELISA kit (Cell Signaling) according to manufacturer’s instructions.

### Western Blot

Whole cell lysates were prepared as previously described^62^. Cell lysates were subjected to SDS-PAGE, transferred to PVDF membranes (Millipore), and then immunoblotted with antibodies against Gapdh (Fitzgerald, #10R-G109a), NF-κBp65 (Cell Signaling, #8242) and phospho-Nf-κBp65 (Cell Signaling, #8033).

### Statistics

Results were analyzed by Student’s t-test (two-tailed) or by ANOVA with Dunnett’s post-test, when multiple comparisons were made. Statistical significance was defined as p < 0.05.

### Data Availability

The microarray datasets generated during and/or analysed during the current study are available in the ArrayExpress database, under accession number E-MTAB-6855.

## Electronic supplementary material

Supplementary Information

Supplementary Tables
