# Author Correction: Adipocytes induce distinct gene expression profiles in mammary tumor cells and enhance inflammatory signaling in invasive breast cancer cells

**DOI:** 10.1038/s41598-024-65268-x

**Published:** 2024-07-01

**Authors:** Annina Nickel, Christina Blücher, Omeir Kadri, Nancy Schwagarus, Silvana Müller, Michael Schaab, Joachim Thiery, Ralph Burkhardt, Sonja C. Stadler

**Affiliations:** 1 Institute of Laboratory Medicine, Clinical Chemistry and Molecular Diagnostics, University Hospital Leipzig, Leipzig, Germany; 2 LIFE Leipzig Research Center for Civilization Diseases, University of Leipzig, Leipzig, Germany

Correction to: *Scientific Reports* 10.1038/s41598-018-27210-w, published online 21 June 2018

Since the publication of this work, Omeir Kadri has changed their name from Omeir Al Kadri. The original article has been corrected.

