# Supplementary material for: Adipocytes induce distinct gene expression profiles in mammary tumor cells and enhance inflammatory signaling in invasive breast cancer cells
Source: Sci Rep. 2018 Jun 21;8:9482. doi: 10.1038/s41598-018-27210-w (PMC6013441; doi:10.1038/s41598-018-27210-w)
Supplement: Supplementary file 1 — Supplementary Information [file 41598_2018_27210_MOESM1_ESM.pdf]

Supplemental Material

Adipocytes induce distinct gene expression profiles in mammary tumor cells and enhance inflammatory signaling in invasive breast cancer cells

Annina Nickel[1#], Christina Blücher[1,2#], Omaeir Al Kadri[1], Nancy Schwagarus[1], Silvana Müller[1], Michael Schaab[1], Joachim Thiery[1,2], Ralph Burkhardt[1,2], Sonja C. Stadler[1,2*]

[1]Institute of Laboratory Medicine, Clinical Chemistry and Molecular Diagnostics, University Hospital Leipzig, Leipzig, Germany

[2]LIFE Leipzig Research Center for Civilization Diseases, University of Leipzig, Leipzig Germany

*Corresponding author: Sonja.Stadler@medizin.uni-leipzig.de

[#]these authors contributed equally

Material and Methods

Oil Red O staining

To visualize the lipid content of undifferentiated and differentiated 3T3-L1 cells, a staining with the lipohilic dye oil red O was performed. 3T3-L1 cells were washed with PBS, fixed with 4% paraformaldehyde (in PBS) for 10 min at RT and washed 2 times with PBS. A oil red O (Sigma-Aldrich, Germany) stock solution (5 g/L in isopropanol) was diluted 6:4 with distilled water and filtered immediately before use. Cells were stained with this oil red O working solution for 20 minutes at RT, followed by two washing steps with 60 % isopropanol and distilled water. Counterstain of the cells was performed by incubating the cells with a hematoxylin staining solution (Sigma-Aldrich, Germany) for 30 sec at RT. Cells were washed 2 times with tap water and PBS and kept in the fridge until imaging. An Axioskope phase contrast microscope with attached AxioCam MRc5 camera (Carl Zeiss AG, Germany) was used to examine the cells. Only plates showing at least 80 % of differentiated 3T3-L1 adipocytes throughout the plate were used for co-culture experiments, as compared to the undifferentiated controls.

IL-6 ELISA

The concentrations of secreted murine IL-6 in cell culture supernatants were determined using murine IL-6 ELISA Kits (R&D Systems) as recommended by the manufacturer.

Supplemental Figures:

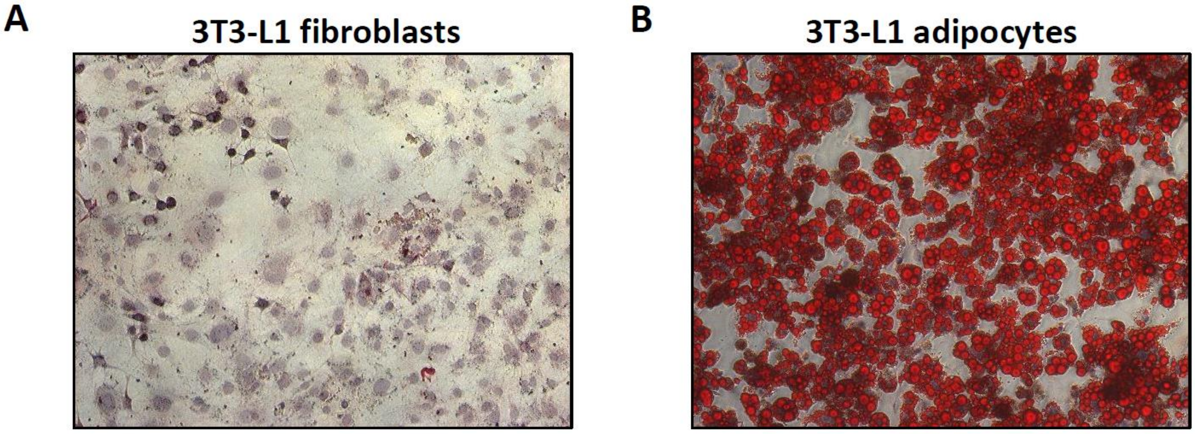

Figure S1: ***In vitro* differentiation of 3T3-L1 preadipocytes into adipocytes.** Representative phase contrast images of oil red O staining (red) of undifferentiated 3T3-L1 preadipocytes (A) and differentiated 3T3-L1 adipocytes (B). Haematoxylin was used as counterstain (blue).

MCF7:

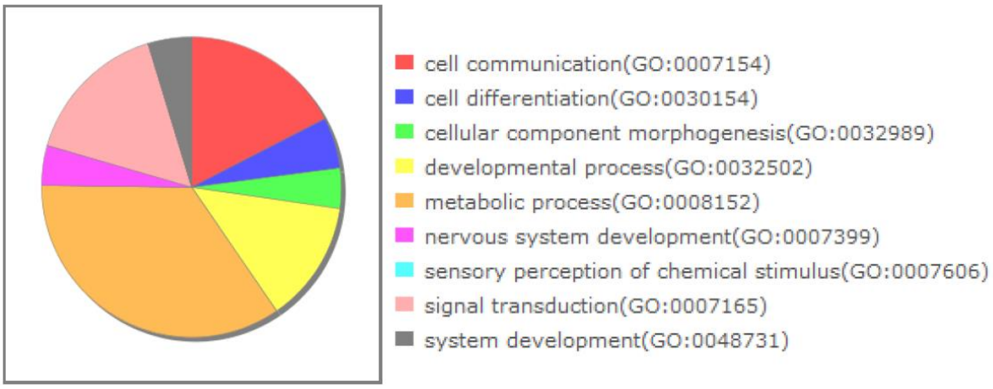

T47D:

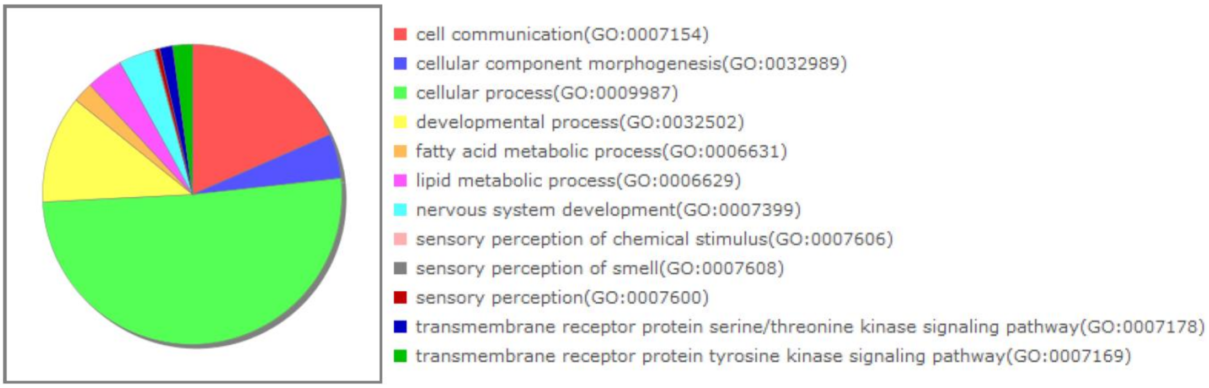

MDA231:

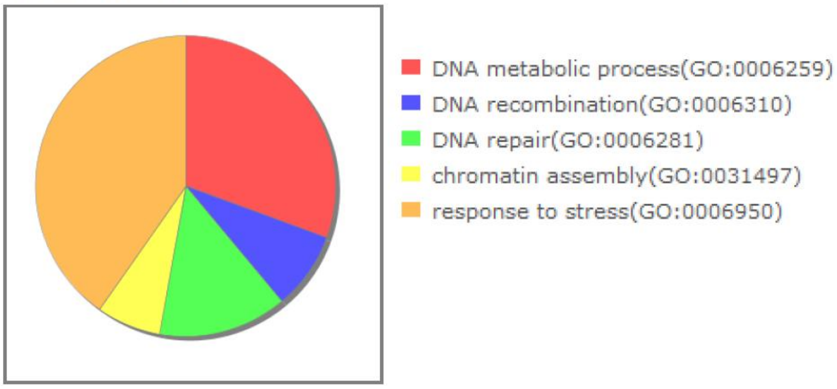

Figure S2: Overrepresentation analysis of GO terms.

Enrichment analysis for GO terms in the PANTHER GO-slim Biological Process data set was carried out using lists of differentially expressed genes from MCF-7, T47D or MDA-MB-231 cells co-cultured with 3T3-L1 adipocytes (≥1.3-fold expression change). The complete homo sapiens gene list was used as reference set to test for overrepresentation. Fisher's exact test with FDR multiple test correction was applied and $p<0.05$ was considered significant.

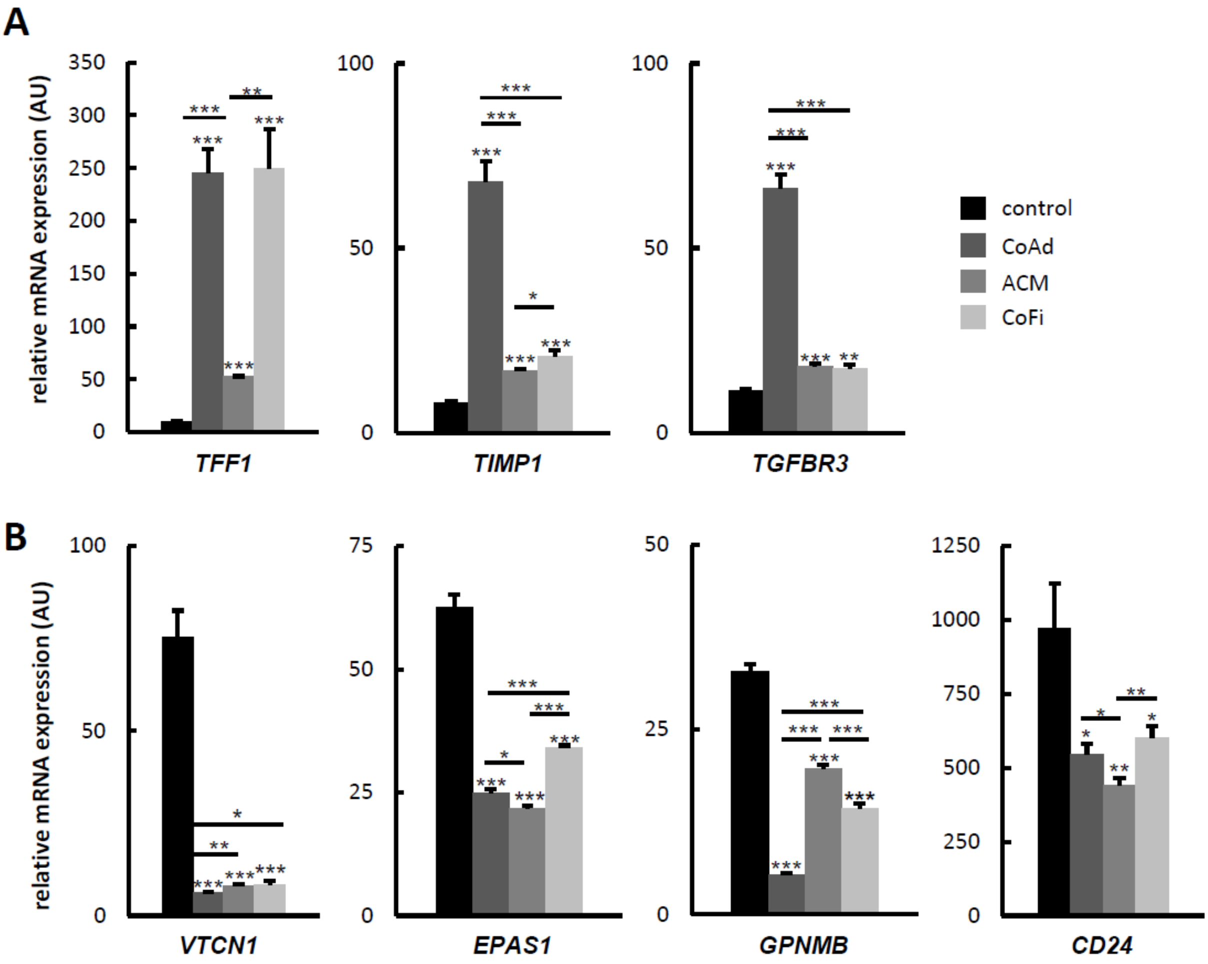

Figure S3: Quantitative RT-PCR analysis of selected candidate genes in T47D cells.

T47D and breast cancer cells were co-cultured either with 3T3-L1 adipocytes (CoAd), 3T3-L1 fibroblasts (CoFi), Adipocyte-conditioned medium (ACM) or alone for 5 days and total RNA was extracted. Relative mRNA expression levels of selected genes found to be differentially expressed in microarray analysis (co-culture with 3T3-L1 adipocytes (CoAd) vs. control) were determined. Data are presented as means ± SD of triplicates from one representative experiment. Significance is relative to control conditions unless otherwise indicated with bars (* $p < 0.05$; ** $p < 0.005$; *** $p < 0.0005$).

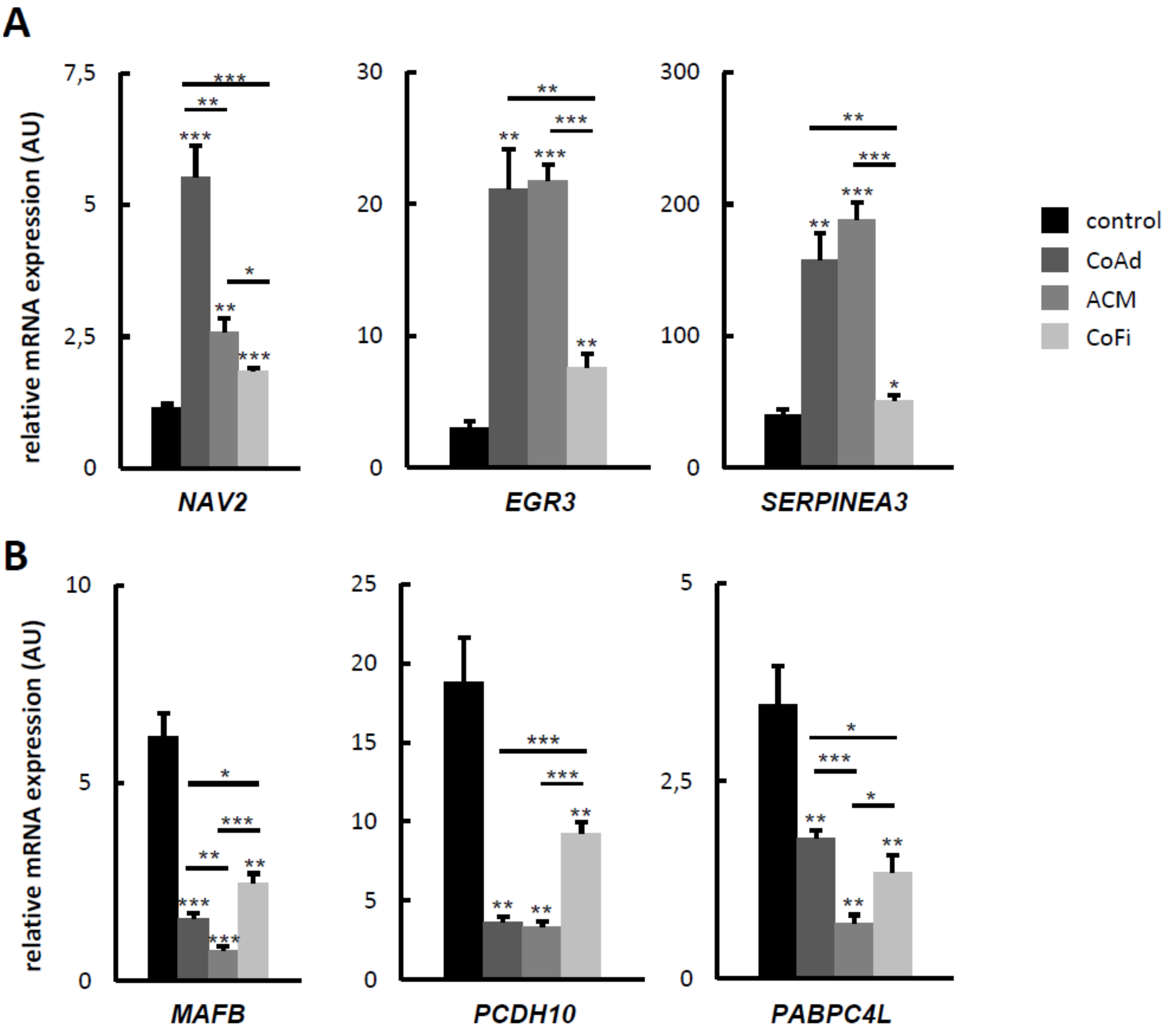

Figure S4: **Validation of selected candidate genes by quantitative RT-PCR in MCF-7 cells.** MCF7 breast cancer cells were co-cultured either with 3T3-L1 adipocytes (CoAd), 3T3-L1 fibroblasts (CoFi), Adipocyte-conditioned medium (ACM) or alone for 5 days prior to total RNA extraction. Relative mRNA expression levels of selected genes found to be differentially expressed in microarray analysis (co-culture with 3T3-L1 adipocytes (CoAd) vs. control) were determined. Data are presented as means ± SD of triplicates from one representative experiment. Significance is relative to basal conditions unless otherwise indicated with bars (* $p < 0.05$; ** $p < 0.005$; *** $p < 0.0005$).

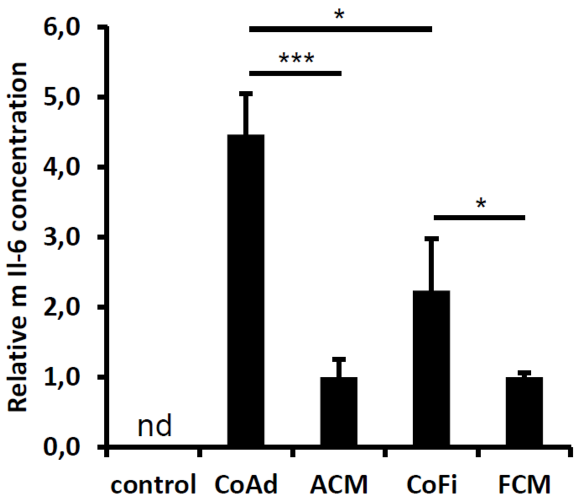

Figure S5: Co-culture with MDA-MB-231 cells enhances IL-6 secretion from adipocytes. Supernatants were collected after 5 days of co-culture between MDA-MB-231 cells and adipocytes (CoAd) or fibroblasts (CoFi), or from MDA-MB-231 cells alone (control). Adipocyte- (ACM) or fibroblast conditioned media (FCM) from differentiated or undifferentiated 3T3-L1 cells were used as controls to assess basal murine IL-6 levels in the media and served as reference. Cytokine expression was determined using an ELISA Kit specifically detecting murine IL-6. Data are presented as means ± SD of triplicates from one representative experiment. Significance is relative to basal media conditions unless indicated otherwise (* $p < 0.05$; ** $p < 0.005$; *** $p < 0.0005$).

To Figure 3b

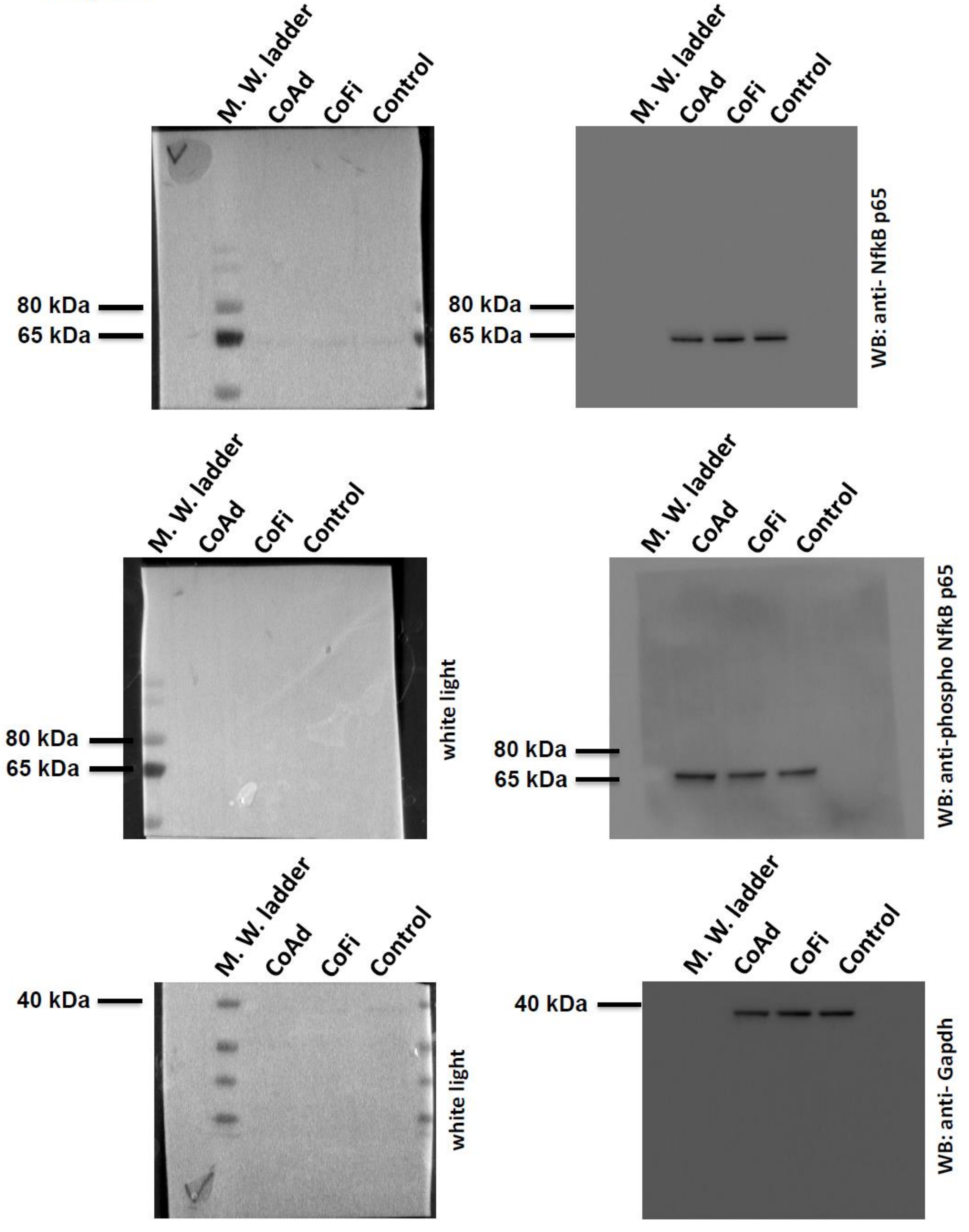

Complete white light and chemiluminescene images of Western Blot shown in Figure 3b.
